# Antibacterial activity of nineteen selected natural products against multi-drug resistant Gram-negative phenotypes

**DOI:** 10.1186/s40064-015-1645-8

**Published:** 2015-12-30

**Authors:** Armelle T. Mbaveng, Louis P. Sandjo, Simplice B. Tankeo, Ache R. Ndifor, Ambassa Pantaleon, Bonaventure T. Nagdjui, Victor Kuete

**Affiliations:** Department of Biochemistry, Faculty of Science, University of Dschang, P.O. Box 67, Dschang, Cameroon; Department of Pharmaceutical Sciences, CSS, Universitade Federal de Santa Catarina, Florianópolis, SC 88040-900 Brazil; Department of Organic Chemistry, Faculty of Science, University of Yaoundé I, Yaoundé, Cameroon; P.O. Box 1499, Bafoussam, Cameroon

**Keywords:** Antibacterial, Flavonoids, Isoflavonoids, Natural products, Multidrug resistance

## Abstract

The present study was designed to assess the antimicrobial activity of 19 natural products belonging to terpenoids, alkaloids, thiophenes and phenolics against a panel of 14 Gram-negative multidrug-resistant (MDR) bacteria. The results demonstrated that amongst the studied compounds, alkaloids and terpenoids were less active contrary to flavonoids: neocyclomorusin (**3**) and candidone (**6**) and isoflavonoids: neobavaisoflavone (**8**) and daidzein (**12**). Thiophene, 2-(penta-1,3-diynyl)-5-(3,4-dihydroxybut-1-ynyl)thiophene (**17**) showed moderate and selective activities. Compounds **3, 6, 8** and **12** displayed minimal inhibitory concentration (MIC) ranged from 4 to 256 μg/mL on all the 14 tested bacteria. MIC values below 10 μg/mL were obtained with **8**, **3**, **6** and **12** against 50, 42.9, 35.7 and 21.4 % of the tested bacteria. The lowest MIC value of 4 μg/mL was obtained with compound **3** against *Klebsiella pneumoniae* ATCC11296, *Enterobacter cloacae* BM47, compound **6** against *Escherichia coli* ATCC8739, *K. pneumoniae* ATCC11296, *E. cloacae* BM47 and compound **8** against *K. pneumoniae* ATCC11296 and *E. cloacae* BM47. The activity of flavonoid **3** was better or equal to that of chloramphenicol in all tested *K. pneumoniae,Providencia stuartii, E. aerogenes, E. cloacae* and *Pseudomonas aeruginosa* strains. Within isoflavonoids, neobavaisoflavone scaffold was detected as a pharmacophoric moiety. This study indicates that natural products such as **3**, **6** and **8** could be explored more to develop antimicrobial drugs to fight MDR bacterial infections.

## Background

Infectious diseases including bacterial infections continue to be a serious health problem worldwide. Multidrug-resistant (MDR) pathogens considerably increase the mortality and morbidity. In effect, clinically, the continuous emergence of Gram-negative MDR bacteria drastically reduced the efficacy of antibiotic arsenal leading globally to an increase of the frequency of therapeutic failure (Rice 2006). Consequently, new antibacterials are needed to fight these bacterial pathogens, but progress in developing them have been slow (Fischbach and Walsh 2009). Plant kingdom represents an enormous source of new chemotherapeutic agents to tackle microbial infections. Several natural compounds belonging to the usual pharmaceutical library have been tested for their ability to combat resistant bacteria (Fischbach and Walsh 2009; Saleem et al. 2010). More than 450 natural metabolites with antimicrobial activity have been reported in the period 2000–2010 (Saleem et al. 2010). Some of the best plant metabolites from African medicinal plants with antibacterial activity against MDR Gram-negative phenotypes include laurentixanthone B (xanthone), diospyrone and plumbagin (naphthoquinone), isobavachalcone and 4-hydroxylonchocarpin (flavonoids) and MAB3 (coumarin) (Kuete et al. 2010, 2011a). The rationale of this work comes to the fact that secondary metabolites belonging to terpenoids, phenolics and alkaloids previously displayed prominent antibacterial activity against MDR Gram-negative bacteria expressing active efflux pumps (Kuete et al. 2010, 2011a). Therefore, the present study was designed to determine the antibacterial activity of several molecules, including terpenoids, alkaloids, thiophenes and phenolics, against different bacterial strains expressing MDR phenotypes. Furthermore, we highlighted the possible pharmacophoric cores amongst the active compounds.

## Results

### Studied compounds

**Fig. 1:**
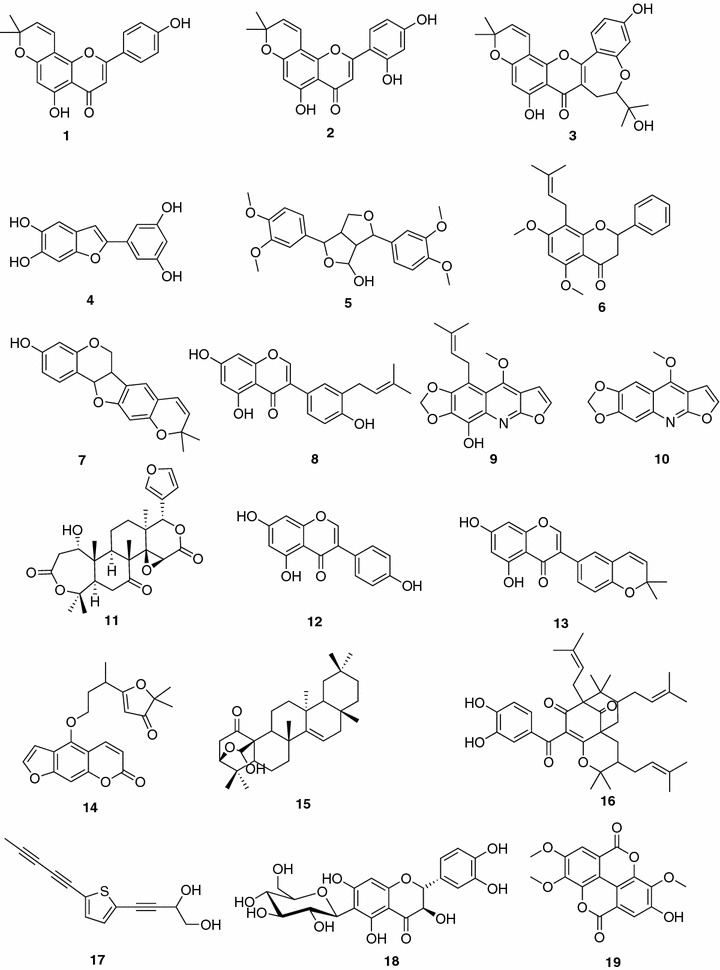
Chemical structures of tested compounds. Atalantoflavone (**1)**; 2′-hydroxyatalantoflavone (**2**); neocyclomorusin (**3**); 2-(3,5-dihydroxyphenyl)benzofuran-5,6-diol (**4**); 4-hydroxy-2,6-di-(3′,4′-dimethoxyphenyl)-3,7-dioxabicyclo-(3.3.0)octane (**5**); Candidone (**6**); isoneorautenol (**7**); neobavaisoflavone (**8**); tecleaverdoornine (**9**); maculine (**10**); deacetylnomilin (**11**); daidzein (**12**); isowighteone (**13**); dorstenin (**14**); herranone (**15**); 2-(penta-1,3-diynyl)-5-(3,4-dihydroxybut-1-ynyl)thiophene (**17**); isogarcinol (**16**); ulmoside A (**18**) and 3,4,3′-tri-*O*-methylellagic acid (**19**)

Compounds tested in the present study (Fig. 1) were previously or newly isolated from several Cameroonian plants. They include atalantoflavone (**1**; yellow solid; m.p. 286.2–287.7 °C) (Ouete et al. 2013); 2′-hydroxyatalantoflavone (**2**; reddish gum) (Ouete et al. 2013); neocyclomorusin (**3**; yellow solid, m.p. 263.2–266.7 °C) (Ouete et al. 2013; Cho et al. 2011); 2-(3,5-dihydroxyphenyl)benzofuran-5,6-diol (**4**; yellow oil) (Ouete et al. 2014; Noguchi et al. 1994); 4-hydroxy-2,6-di-(3′,4′-dimethoxyphenyl)-3,7-dioxabicyclo-(3.3.0)octane (**5**; yellow solid; m.p. 168.5–169.9 °C) (Kuete et al. 2013); candidone (**6**; yellowish powder; m.p. 95.1–96.2 °C) (Ouete et al. 2013; Kuete et al. 2013); isoneorautenol (**7**; yellowish solid; m.p. 156.2–157.8 °C) (Nkengfack et al. 1995; Kuete et al. 2014); neobavaisoflavone (**8**; yellowish oil) (Nkengfack et al. 1994; Kuete et al. 2014); tecleaverdoornine (**9**; amorphous solid) (Ayafor and Okogun 1982; Sandjo et al. 2014); maculine (**10**; amorphous solid) (Nunes et al. 2005; Kuete et al. 2008b; Sandjo et al. 2014); deacetylnomilin (**11**; colorless oil) (Bennett and Hasegawa 1981); daidzein (**12**; yellowish gum) (Basha et al. 2013); isowighteone (**13**; yellowish gum) (Wang et al. 2001); dorstenin (**14**; white solid; m.p. 136–139 °C) (Kuster et al. 1994; Abegaz et al. 2004); herranone (**15**; colorless solid; m.p. 285–287 °C) (Wiedemann et al. 1999); isogarcinol (**16**; brown oil) (Marti et al. 2010); 2-(penta-1,3-diynyl)-5-(3,4-dihydroxybut-1-ynyl)thiophene (**17**; brownish oil) (Shi et al. 2010); ulmoside A (**18**; brown oil) (Rawat et al. 2009) and 3,4,3′-tri-*O*-methylellagic acid (**19**; brown solid; m.p. 282–284 °C) (Gao et al. 2014). These compounds belong to flavonoids (**1-3, 6, 18**), isoflavonoids (**7, 8, 12, 13**), benzophenone (**16**), benzofuran (**4**), ellagic acid derivative (**19**), lignan (**5**), alkaloids (**9, 10**), terpenoids (**11, 15**) and thiophene (**19**). They were tested for their antimicrobial activity on a panel of 14 bacterial strains and the results are summarized in Table 1.

**Table 1:** MICs and MBCs (µg/mL) of the nineteen tested compounds and ciprofloxacin on the panel of tested bacteria

Samples	Bacterial strains, MIC and MBC (µg/mL)													
	*Escherichia coli*			*Enterobacter aerogenes*			*Klebsiella pneumoniae*		*Providencia stuartii*		*Enterobacter* *cloacae*		*Pseudomonas aeruginosa*	
	ATCC 8739	AG102	AG100 A_tet_	ATCC 13048	CM64	EA27	ATCC 11296	KP55	ATCC29916	PS299645	BM47	BM67	PA01	PA124
**1**	256 (–)	–	–	256 (–)	–	–	–	–	–	128 (–)	–	–	–	–
**2**	256 (–)	–	–	256 (–)	–	–	–	–	–	256 (–)	–	–	–	–
**3**	**8** (16)	32 (128)	32 (128)	**8** (16)	32 (128)	16 (64)	**4** (**8**)	**8** (16)	**8** (32)	32 (64)	**4** (**8**)	16 (128)	32 (128)	64 (256)
**4**	256 (–)	256 (–)	–	256 (–)	256 (–)	256 (–)	–	–	256 (–)	256 (–)	256 (–)	–	–	–
**5**	–	–	–	–	–	–	–	–	–	256 (–)	–	–	–	–
**6**	**4** (32)	**8** (16)	256 (–)	256 (–)	256 (–)	16 (32)	**4** (**8**)	**8** (16)	16 (128)	64 (256)	**4**(**8**)	256 (–)	64 (256)	256 (–)
**7**	256 (–)	–	–	256 (–)	–	–	–	–	–	–	–	256 (–)	–	–
**8**	**8** (32)	16 (64)	256 (–)	128 (–)	256 (–)	32 (128)	**8** (16)	**8** (32)	**4** (16)	**8** (32)	**4** (16)	256 (–)	**8** (32)	64 (256)
**9**	256 (–)	–	–	–	–	–	–	–	–	–	–	–	–	–
**10**	256 (–)	–	–	256 (–)	–	–	–	–	–	–	–	–	–	–
**11**	256 (–)	–	–	–	–	–	–	–	256 (–)	–	–	–	–	–
**12**	64 (128)	128 (–)	128 (–)	256 (–)	256 (–)	128 (–)	128 (256)	128 (–)	64 (256)	128 (–)	64 (128)	256 (–)	128 (256)	256 (–)
**13**	128 (–)	–	128 (–)	256 (–)	–	–	–	–	–	256 (–)	–	–	–	–
**14**	–	–	–	256 (–)	–	256 (–)	–	–	–	256 (–)	256 (–)	256 (–)	–	–
**15**	128 (–)	–	–	256 (–)	–	–	–	–	–	128 (–)	256 (–)	256 (–)	–	–
**16**	–	–	–	–	–	–	–	–	–	–	–	–	–	–
**17**	64 (128)	128 (–)	256 (–)	64 (256)	256 (–)	64 (256)	64 (256)	128 (–)	64 (256)	256 (–)	256 (–)	–	256 (–)	–
**18**	–	–	–	256 (–)	–	–	–	–	–	256 (–)	–	–	–	–
**19**	64 (256)	256 (–)	256 (–)	128 (–)	256 (–)	256 (–)	64 (256)	256 (–)	16 (64)	32 (256)	256 (–)	256 (–)	–	–
**CHL**	**4** (64)	**8** (–)	**8** (–)	16 (128)	–	128 (–)	16 (128)	128 (–)	**8** (128)	32 (–)	–	256 (–)	16 (256)	64 (256)

*CHL* chloramphenicol, (–) MIC > 256 µg/mL, *in bold* significant antibacterial activity (Kuete 2010; Kuete and Efferth 2010)

Atalantoflavone (**1**); 2′-hydroxyatalantoflavone (**2**); neocyclomorusin (**3**); 2-(3,5-dihydroxyphenyl)benzofuran-5,6-diol (**4**); 4-hydroxy-2,6-di-(3′,4′-dimethoxyphenyl)-3,7-dioxabicyclo-(3.3.0)octane (**5**); Candidone (**6**); isoneorautenol (**7**); neobavaisoflavone (**8**); tecleaverdoornine (**9**); maculine (**10**); deacetylnomilin (**11**); daidzein (**12**); isowighteone (**13**); dorstenin (**14**); herranone (**15**); 2-(penta-1,3-diynyl)-5-(3,4-dihydroxybut-1-ynyl)thiophene (**17**); isogarcinol (**16**); ulmoside A (**18**) and 3,4,3′-tri-*O*-methylellagic acid (**19**). Compound classes [flavonoids (**1-3, 6, 18**), isoflavonoids (**7, 8, 12, 13**), benzophenone (**16**), benzofuran (**4**), ellagic acid derrivative (**19**), lignan (**5**), alkaloids (**9, 10**), terpenoids (**11, 15**) and thiphene (**19**)]

Bold values indicate Significant activity

### Activity of terpenoids

Both diterpenoid (**11**) and triterpenoid (**15**) exhibited very weak activities. Deacetylnomilin (**11**) and herranone (**15**) had detectable MIC values against 2/14 (14.3 %) and 5/14 (35.7 %) bacterial strains respectively (Table 1). However, no minimal bactericidal concentration (MBC) value was obtained with the two terpenoids.

### Activity of alkaloids

Furoquinoline alkaloids tecleaverdoornine (**9**) and maculine (**10**) displayed low activity against the tested bacteria. Their inhibitory effects were observed on 1/14 (7.1 %) and 2/14 (14.3 %) microbial strains, respectively, for **9** and **10** (Table 1).

### Activity of thiophene

The compound 2-(penta-1,3-diynyl)-5-(3,4-dihydroxybut-1-ynyl)thiophene (**17**) showed MIC values below 100 µg/mL against *Escherichia coli* ATCC 8739, *Enterobacter aerogenes* ATCC 13048 and EA27, *Klebsiella pneumoniae* ATCC11296 and *Providencia stuartii* ATCC29916 (Table 1).

### Activity of phenolics (flavonoids, isoflavonoids, benzophenone, benzofuran, coumarins, ellagic acid derrivative and lignan)

The best activities were obtained with phenolic compounds; amongst them, flavonoids neocyclomorusin (**3**) and candidone (**6**) as well as isoflavonoids neobavaisoflavone (**8**) and daidzein (**12**) had MIC values ranged from 4 to 256 µg/mL on all the 14 tested bacteria. Moreover, MIC values below 10 µg/mL were obtained with **8**, **3**, **6** and **12** against 7/14 (50 %), 6/14 (42.9 %), 5/14 (35.7 %) and 3/14 (21.4 %) tested bacteria respectively. MIC values below 100 µg/mL were obtained with compound **3** on all tested bacteria. MBC values below 10 µg/mL were also obtained with compound **3** against *K. pneumoniae* ATCC11296 and *Enterobacter cloacae* BM47 whilst values ranged from 8 to 256 µg/mL were noted on all tested pathogens. The lowest MIC value of 4 µg/mL was obtained with compound **3** against *K. pneumoniae* ATCC11296, *E. cloacae* BM47, compound **6** against *E. coli* ATCC8739, *K. pneumoniae* ATCC11296, *E. cloacae* BM47 and compound **8** against *K. pneumoniae* ATCC11296 and *E. cloacae* BM47.

### Structure–activity relationship study

**Fig. 2:**
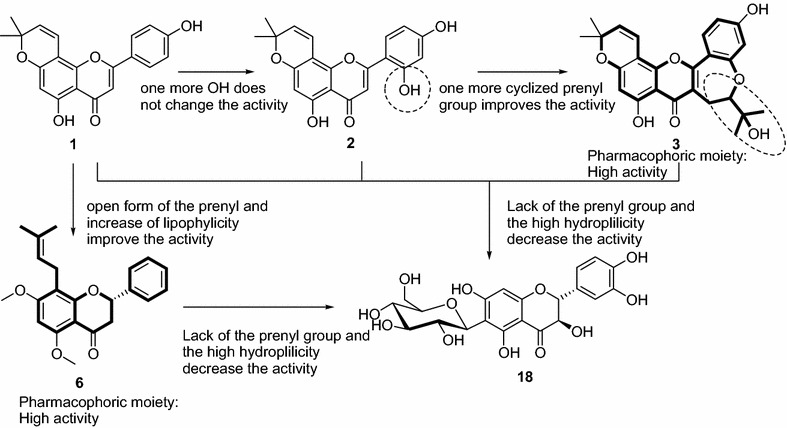
Pharmacophores (**3** and **6**) detected in flavonoids

**Fig. 3:**
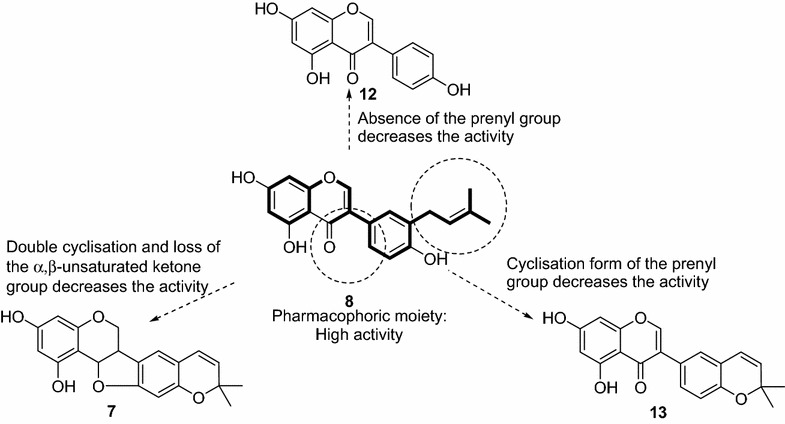
Representation of pharmacophoric scaffold detected in isoflavonoids

When analyzing the structure–activity relationship, it can be observed that terpenoids (both diterpenoids and triterpenoids) as well as the tested furoquinoline alkaloids were poor antimicrobial compounds. A keen look of the activities of phenolics shows that benzophenone (**16**) were not active meanwhile coumarin (**14**), lignan (**5**) and benzofuran (**4**) were also found to be poor antibacterial agents. The best activities were obtained with flavonoids and isoflavonoids. Within flavonoids, it appeared that hydroxylation of compound **1** to yield **2** did not significantly changed the antibacterial activity (Table 1). The presence of a cyclic prenyl moiety in the heterocyclic portion of flavone **3** improves significantly the activity and provides a large antimicrobial selectivity. Regardless the presence of the α,β-unsaturated double bond, compound **6** showed interesting activity like **3.** Compound **6** differs from **1** to **3** with the α,β-unsaturated double bond and its high lipophilicity turning this latter along with **3** as two pharmacophores to be explored for antimicrobial chemotherapy. Nevertheless, the high hydrophilicity of compound **18** reduced drastically its antimicrobial potency (Fig. 2). Concerning the activity of isoflavonoid, it also appeared that neobavaisoflavone (**8**) was another pharmacophoric moiety (Fig. 3). Any modification of the structure of compound **8** such as cyclisation or absence of prenyl group resulted in the reduction of antibacterial activity as it can be seen with compounds **7, 12** and **13**.

## Discussion

Bacterial multidrug resistance represents a major hurdle in the treatment of infectious diseases. In the present study, we tested a panel of bacterial strains including both reference ATCC strains and MDR phenotypes expressing active efflux pumps (Kuete et al. 2010, 2011a; Fankam et al. 2011). In fact, tripartite drug efflux pumps, mainly those clinically reported as AcrAB–TolC in Enterobacteriaceae or as MexAB–OprM in *Pseudomonas aeruginosa* tested in the present study, play a key role in multidrug resistance of pathogenic Gram-negative bacteria (Nikaido 2009; Davin-Regli et al. 2008). Interestingly, a MIC value of 64 µg/mL was recorded with the best compounds (namely flavonoid **3** and isoflavonoid **8)** against the problematic MDR strain *P. aeruginosa* PA124. This value was identical to that of the reference compound chloramphenicol (Table 1). The antimicrobial activity of a phytochemical has been defined as significant when MIC is below 10 µg/mL, moderate when 10 µg/mL < MIC < 100 µg/mL or low when MIC > 100 µg/mL (Kuete 2010; Kuete and Efferth 2010). In the present study, MIC values below 10 µg/mL were noted with compounds **3**, **6** and **8** against several bacterial strains, highlighting their possible use in the control of bacterial infections. The activity of flavonoid **3** was better or equal to that of chloramphenicol in the majority of the bacteria including all tested *K. pneumoniae, P. stuartii, E. aerogenes, E. cloacae* and *P. aeruginosa* strains (Table 1). This was also the case with compounds **6** and **8** towards the majority of the tested bacterial strains and mostly the MDR phenotypes. Regarding the involvement of MDR bacteria in treatment failures and the re-emergence of infectious diseases (Blot et al. 2007; Falagas and Bliziotis 2007; Nicolle 2001), the activity of flavonoids **3, 6** and isoflavonoid **8** could be considered very promising. *Pseudomonas aeruginosa* is an important nosocomial pathogen highly resistant to clinically used antibiotics, causing a wide spectrum of infections and leading to substantial morbidity and mortality (Cardoso et al. 2007) and was found sensitive to the three compounds (**3, 6** and **8**). MDR Enterobacteriaceae, including *K. pneumoniae*, *E. aerogenes, P. stuartii* and *E. coli*, have also been classified as antimicrobial-resistant organisms of concern in healthcare facilities (Nicolle 2001; Tran et al. 2010). The analysis of data of Table 1 shows that MBC/MIC ratios below 4 were recorded with the three best compounds (**3, 6** and **8**) in many cases, suggesting that bacterial effects of these phytochemicals could be expected (Mims et al. 1993; Mbaveng et al. 2011, 2012). The data reported herein highlight once more the good pharmacological potential of flavonoids **3, 6** and isoflavonoid **8** and their ability to combat infections involving these bacterial species. Furthermore, the pharmacophoric moiety (**8**) suggests that hemi-synthesis reaction with neobavaisoflavone could be explored in more details for antibacterial drug development. To the best of our knowledge, the antibacterial activity of the best compounds (**3, 6** and **8**) against MDR bacteria is being reported for the first time. However, several flavonoids and isoflavonoids are known to possess antibacterial activities against both drug-sensitive and MDR Gram-negative phenotypes (Kuete et al. 2010, 2011a; Ndhlala et al. 2013; Ngameni et al. 2013).

## Methods

### General procedure

Column chromatography (CC) and thin layer chromatography (TLC) were performed over silica gel 60H (particle size 90 % <45 mm), 200–300 mesh silica gel silica gel GF254, respectively. 1D- and 2D-NMR spectra were carried out with a Bruker DRX-400 MHz. Melting points were measured by an Electro thermal IA 9000 digital melting point apparatus and are uncorrected.

### Plant material

Plant species were collected in Yaoundé (Centre Region, Cameroon) and in Dschang (West Region, Cameroon) and identified by the specialist of the national herbarium in Yaoundé, Cameroon where their voucher are kept under the registration codes: *Teclea afzelii* Engl. (Rutaceae) (10674/SRF/Cam), *Erythrina excelsa* Baker (Fabaceae) (61487/HNC), *Erythrina senegalensis* A. Rich. (Fabaceae) (59409/HNC)*, Echinops giganteus* A. Rich. (Asteraceae) (23647/SRF-Cam)*, Pachystela msolo* Engl. (Sapotaceae) (3849/SRFK)*, Garcinia ovalifolia* Oliv. (Guttiferae) (55523/HNC) and *Alchornea laxiflora* (Benth) Pax and Hoff (Euphorbiaceae) (45363HNC).

### Ethics statement

For the collection of plants, no specific permits were required for the described field studies. For any location/activity, no specific permissions were required. All locations where the plants were collected were not privately-owned or protected in any way and the field studies did not involved endangered or protected species.

### Extraction and isolation

Compounds tested in the present work were either previously isolated in our research team (**1–10, 14**) or newly isolated (**12, 13, 15–19**). Compounds **1**–**3** were obtained from the roots of *Milicia excels*a whilst **4** was identified from the leaves as previously reported (Ouete et al. 2013, 2014). Compounds **5** and **6** were isolated from *Echinops giganteus* (Asteraceae) (Kuete et al. 2013), while **7** and **8** were obtained from *Erythrina sigmoidea* (Fabaceae) (Kuete et al. 2014). Compounds **9** and **10** were isolated from *Zanthoxylum buesgenii* (Rutaceae) (Sandjo et al. 2014). Compound **14** was isolated from *Dorsteniaelliptica* (Moraceae) (Abegaz et al. 2004).

Compound **11** in addition to **9** and **10** were isolated from the roots of *Teclea afzelii* (Rutaceae); Hence, air-dried roots (7 kg) were macerated in MeOH/DCM (1:1, v/v) for 48 h and the organic (286 g) solid obtained after evaporation of the solvents *in vacuo* was further extracted with hexane (hex, 41 g), ethyl acetate (EA, 44 g) and MeOH (201 g). The EA fraction was purified by column chromatography on silica gel in gradient conditions of hex/EA. Three compounds were isolated as follows: **9** (6 mg), **10** (5 mg) and **11** (10 mg).

Compound **12** was obtained from the roots of *Erythrina excelsa* (2.2 kg) (Fabaceae); The crude extract obtained from the maceration of the air-dried roots was fractioned by silica gel flash chromatography using hex/EA in gradient conditions. The fraction issued from hex/EA (1:1) was chromatographed using the same condition as above from which **12** (45 mg) was isolated. Similarly, **13** (5 mg) was isolated from the roots of *Erythrina senegalensis* (Fabaceae) as described for **12**. Compound **17** (5.0 mg) was isolated from the roots of *Echinops giganteus*; the powdered roots of *E. giganteus* (Asteraceae) was macerated successively in DCM/MeOH (1:1, v/v) and MeOH for 48 h and 24 h, respectively. The organic solutions were pooled together based on their TLC profile. Eighty-one grams of a red dark crude extract were obtained after evaporation *invacuo*. Furthermore, the crude extract was poured onto water and extracted with Hex (A, 10 g), DCM (B, 25 g), EA (C, 30 g), *n*-butanol (D, 5 g). Fraction B was purified on silica gel CC using gradient conditions of Hex/EA and **17** was obtained from the Hex/EA (3:2, v/v). Similarly, bark powder (2.8 kg) of *Pachystela msolo* Engl. (Sapotaceae) was extracted with DCM/MeOH (1:1), yielding a dark crude extract (30 g). The purification of this later in gradient conditions of Hex/EA afforded **15** (5 mg). The stem bark (2.5 kg) of *Garcinia ovalifolia* (Guttiferaceae) was air-dried, ground and macerated in MeOH for 48 h. A brown residue (120 g) was obtained after concentrating the organic solution. Vacuum liquid chromatography was used for a first fractionation with gradient of hex/EA and EA/MeOH. Fractions obtained from Hex and Hex/EA (3:1, v/v) were pooled together (1.22 g) and purified by silica gel CC with gradients of the same mixture of solvents to afford **16** (12 mg). Fractions collected from EA/MeOH (9:1 and 4:1) were also pooled together and purified on silica gel CC with gradients of DCM/MeOH to yield compound **18** (90 mg). The stem bark (1.8 kg) of *Alchornea Laxiflora* (Euphorbiaceae) was macerated in MeOH. The concentrated methanol crude extract (30 g) was subjected to silica gel flash chromatography using Hex (A), EA (B) and MeOH (C). Fraction C (10 g) was purified by silica gel CC using the gradient of DCM/MeOH. Compound **19** (15 mg) was isolated from sub-fractions eluted with DCM/MeOH (95:5).

### Antimicrobial assays

#### Chemicals for antimicrobial assay

Chloramphenicol ≥98 % (Sigma-Aldrich, St. Quentin Fallavier, France) was used as reference antibiotics (RA) against Gram-negative bacteria. *p*-Iodonitrotetrazolium chloride ≥97 % (INT, Sigma-Aldrich) was used as microbial growth indicator (Eloff 1998; Mativandlela et al. 2006).

#### Microbial strains and culture media

The studied microorganisms included sensitive and resistant strains of *P. aeruginosa, K. pneumoniae, E. aerogenes, E. cloacae, E. coli, P. stuartii,* obtained from the American Type Culture Collection. Their bacterial features were previously reported (Kuete et al. 2011a; Lacmata et al. 2012; Seukep et al. 2013; Touani et al. 2014). Nutrient agar were used for the activation of the tested Gram-negative bacteria (Kuete et al. 2011b).

#### INT colorimetric assay for MIC and MBC determinations

The minimal inhibitory concentration (MIC) determinations on the tested bacteria were conducted using rapid *p*-iodonitrotetrazolium chloride (INT) colorimetric assay according to described methods (Eloff 1998) with some modifications (Kuete et al. 2008b, 2009). The test samples and RA were first of all dissolved in DMSO/Mueller–Hinton Broth (MHB). The final concentration of DMSO was lower than 2.5 % and does not affect the microbial growth (Kuete et al. 2007, 2008a). The solution obtained was then added to Mueller–Hinton Broth, and serially diluted two fold (in a 96-wells microplate). One hundred microlitre (100 µL) of inoculum 1.5 × 10^6^ CFU/mL prepared in appropriate broth was then added (Kuete et al. 2008b, 2009). The plates were covered with a sterile plate sealer, then agitated to mix the contents of the wells using a plate shaker and incubated at 37 °C for 18 h. The assay was repeated thrice. Wells containing adequate broth, 100 µL of inoculum and DMSO to a final concentration of 2.5 % served as negative control. The MIC of samples was detected after 18 h incubation at 37 °C, following addition (40 µL) of 0.2 mg/mL of INT and incubation at 37 °C for 30 min. Viable bacteria reduced the yellow dye to a pink. MIC was defined as the sample concentration that prevented the color change of the medium and exhibited complete inhibition of microbial growth (Eloff 1998). The minimal bactericidal concentration (MBC) was determined by adding 50 µL aliquots of the preparations, which did not show any growth after incubation during MIC assays, to 150 µL of adequate broth. These preparations were incubated at 37 °C for 48 h. The MBC was regarded as the lowest concentration of extract, which did not produce a color change after addition of INT as mentioned above (Kuete et al. 2008b, 2009).

## Conclusion

Regarding the medical importance of the studied microorganisms, the results obtained and reported in this study interestingly showed how secondary metabolites are still a strong source of inspiration in drug discovery. Thus, the present data provided evidence that neocyclomorusin (**3**), candidone (**6**) and neobavaisoflavone (**8**) could be potential antimicrobial drugs to fight MDR bacterial infections and could also be used as motifs for developing related antibiotics with strong potency. To explore more the therapeutic values of the studied compounds, a combination with commonly used antibiotics will be further performed. Also, the study of the mechanism of action of the effective compounds will be carried out to better understand their inhibitory effects.

## References

[CR1] Abegaz BM, Ngadjui BT, Folefoc GN, Fotso S, Ambassa P, Bezabih M, Dongo E, Rise F, Petersen D (2004). Prenylated flavonoids, monoterpenoid furanocoumarins and other constituents from the twigs of *Dorstenia elliptica* (Moraceae). Phytochemistry.

[CR2] Ayafor J, Okogun J (1982). Isolation and identification of three new phenolic furoquinoline alkaloids from *Teclea verdoorniana* Exell and Mendonça (Rutaceae). J Chem Soc Perkin Trans.

[CR3] Basha G, Yadav S, Srinuvasarao R, Prasanthi S, Ramu T, Mangarao N, Siddaiah V (2013). A mild and efficient protocol to synthesize chromones, isoflavones, and homoisoflavones using the complex. Can J Chem.

[CR4] Bennett R, Hasegawa S (1981). Limonoids of calamondin seeds. Tetrahedron.

[CR5] Blot S, Depuydt P, Vandewoude K, De Bacquer D (2007). Measuring the impact of multidrug resistance in nosocomial infection. Curr Opin Infect Dis.

[CR6] Cardoso O, Alves AF, Leitao R (2007). Surveillance of antimicrobial susceptibility of *Pseudomonas aeruginosa* clinical isolates from a central hospital in Portugal. J Antimicrob Chemother.

[CR7] Cho JK, Ryu YB, Curtis-Long MJ, Kim JY, Kim D, Lee S, Lee WS, Park KH (2011). Inhibition and structural reliability of prenylated flavones from the stem bark of *Morus lhou* on beta-secretase (BACE-1). Bioorg Med Chem Lett.

[CR8] Davin-Regli A, Bolla JM, James CE, Lavigne JP, Chevalier J, Garnotel E, Molitor A, Pages JM (2008). Membrane permeability and regulation of drug “influx and efflux” in enterobacterial pathogens. Curr Drug Targets.

[CR9] Eloff JN (1998). A sensitive and quick microplate method to determine the minimal inhibitory concentration of plant extracts for bacteria. Planta Med.

[CR10] Falagas ME, Bliziotis IA (2007). Pandrug-resistant Gram-negative bacteria: the dawn of the post-antibiotic era?. Int J Antimicrob Agents.

[CR11] Fankam AG, Kuete V, Voukeng IK, Kuiate JR, Pages JM (2011). Antibacterial activities of selected Cameroonian spices and their synergistic effects with antibiotics against multidrug-resistant phenotypes. BMC Complement Altern Med.

[CR12] Fischbach MA, Walsh CT (2009). Antibiotics for emerging pathogens. Science.

[CR13] Gao X, Wu J, Zou W, Dai Y (2014). Two ellagic acids isolated from roots of *Sanguisorba officinalis* L. promote hematopoietic progenitor cell proliferation and megakaryocyte differentiation. Molecules.

[CR14] http://whqlibdoc.who.int/hq/2001/WHO_CDS_CSR_DRS_2001.7.pdf. Accessed October 2014

[CR15] Kuete V (2010). Potential of Cameroonian plants and derived products against microbial infections: a review. Planta Med.

[CR16] Kuete V, Efferth T (2010). Cameroonian medicinal plants: pharmacology and derived natural products. Front Pharmacol.

[CR17] Kuete V, Wabo GF, Ngameni B, Mbaveng AT, Metuno R, Etoa FX, Ngadjui BT, Beng VP, Meyer JJ, Lall N (2007). Antimicrobial activity of the methanolic extract, fractions and compounds from the stem bark of *Irvingia gabonensis* (Ixonanthaceae). J Ethnopharmacol.

[CR18] Kuete V, Ngameni B, Simo CC, Tankeu RK, Ngadjui BT, Meyer JJ, Lall N, Kuiate JR (2008). Antimicrobial activity of the crude extracts and compounds from *Ficus chlamydocarpa* and *Ficus cordata* (Moraceae). J Ethnopharmacol.

[CR19] Kuete V, Wansi JD, Mbaveng AT, Kana Sop MM, Tadjong AT, Beng VP, Etoa FX, Wandji J, Meyer JJM, Lall N (2008). Antimicrobial activity of the methanolic extract and compounds from *Teclea afzelii* (Rutaceae). S Afr J Bot.

[CR20] Kuete V, Nana F, Ngameni B, Mbaveng AT, Keumedjio F, Ngadjui BT (2009). Antimicrobial activity of the crude extract, fractions and compounds from stem bark of *Ficus ovata* (Moraceae). J Ethnopharmacol.

[CR21] Kuete V, Ngameni B, Tangmouo JG, Bolla JM, Alibert-Franco S, Ngadjui BT, Pages JM (2010). Efflux pumps are involved in the defense of Gram-negative bacteria against the natural products isobavachalcone and diospyrone. Antimicrob Agents Chemother.

[CR22] Kuete V, Alibert-Franco S, Eyong KO, Ngameni B, Folefoc GN, Nguemeving JR, Tangmouo JG, Fotso GW, Komguem J, Ouahouo BM, Bolla JM, Chevalier J, Ngadjui BT, Nkengfack AE, Pages JM (2011). Antibacterial activity of some natural products against bacteria expressing a multidrug-resistant phenotype. Int J Antimicrob Agents.

[CR23] Kuete V, Kamga J, Sandjo LP, Ngameni B, Poumale HM, Ambassa P, Ngadjui BT (2011). Antimicrobial activities of the methanol extract, fractions and compounds from *Ficus polita* Vahl. (Moraceae). BMC Complement Altern Med.

[CR24] Kuete V, Sandjo L, Wiench B, Efferth T (2013). Cytotoxicity and modes of action of four Cameroonian dietary spices ethno-medically used to treat Cancers: *Echinops giganteus,* Xylopia aethiopica, *Imperata cylindrica* and *Piper capense*. J Ethnopharmacol.

[CR25] Kuete V, Sandjo LP, Djeussi DE, Zeino M, Kwamou GM, Ngadjui B, Efferth T (2014). Cytotoxic flavonoids and isoflavonoids from *Erythrina sigmoidea* towards multi-factorial drug resistant cancer cells. Invest New Drugs.

[CR26] Kuster R, Robson R, Bernardo R, Da Silva A, Parente J, Mors W (1994). Furocoumarins from the rhizomes of *Dorstenia brasiliensis*. Phytochemistry.

[CR27] Lacmata ST, Kuete V, Dzoyem JP, Tankeo SB, Teke GN, Kuiate JR, Pages JM (2012). Antibacterial activities of selected Cameroonian plants and their synergistic effects with antibiotics against bacteria expressing MDR phenotypes. Evid Based Complement Alternat Med.

[CR28] Marti G, Eparvier V, Litaudon M, Grellier P, Guéritte F (2010). A new xanthone from the bark extract of *Rheedia acuminata* and antiplasmodial activity of its major compounds. Molecules.

[CR29] Mativandlela SPN, Lall N, Meyer JJM (2006). Antibacterial, antifungal and antitubercular activity of (the roots of) *Pelargonium reniforme* (CURT) and *Pelargonium sidoides* (DC) (Geraniaceae) root extracts. S Afr J Bot.

[CR30] Mbaveng AT, Kuete V, Mapunya BM, Beng VP, Nkengfack AE, Meyer JJ, Lall N (2011). Evaluation of four Cameroonian medicinal plants for anticancer, antigonorrheal and antireverse transcriptase activities. Environ Toxicol Pharmacol.

[CR31] Mbaveng AT, Kuete V, Ngameni B, Beng VP, Ngadjui BT, Meyer JJ, Lall N (2012). Antimicrobial activities of the methanol extract and compounds from the twigs of *Dorstenia mannii* (Moraceae). BMC Complement Altern Med.

[CR32] Mims C, Playfair J, Roitt I, Wakelin D, Williams R (1993) Antimicrobials and chemotherapy. In: Mims CA, et al (eds) Med Microbiol Rev, vol. 35, pp 1–34

[CR33] Ndhlala AR, Amoo SO, Ncube B, Moyo M, Nair JJ, Van Staden J, Kuete V (2013). 16—antibacterial, antifungal, and antiviral activities of African medicinal plants. Medicinal plant research in Africa: Pharmacology and Chemistry.

[CR34] Ngameni B, Fotso GW, Kamga J, Ambassa P, Abdou T, Fankam AG, Voukeng IK, Ngadjui BT, Abegaz BM, Kuete V, Kuete V (2013). 9—Flavonoids and related compounds from the medicinal plants of Africa. Medicinal Plant Research in Africa: Pharmacology and Chemistry.

[CR35] Nicolle L (2001) Infection control programmes to contain antimicrobial resistance. World Health Organization, Geneva **(WHO/CDS/CSR/DRS/20017)**

[CR36] Nikaido H (2009). Multidrug resistance in bacteria. Annu Rev Biochem.

[CR37] Nkengfack AE, Vouffo TW, Fomum ZT, Meyer M, Bergendorff O, Sterner O (1994). Prenylated isoflavanone from the roots of *Erythrina sigmoidea*. Phytochemistry.

[CR38] Nkengfack AE, Vardamides JC, Fomum ZT, Meyer M (1995). Prenylated isoflavanone from *Erythrina eriotricha*. Phytochemistry.

[CR39] Noguchi A, Yoshihara T, Ichihara A, Sugihara S, Koshino M, Kojima M, Masaoka Y (1994). Ferric phosphate-dissolving compound, alfafuran, from alfalfa (*Medicago sativa* L.) in response to iron-deficiency stress. Biosci Biotechnol Biochem.

[CR40] Nunes F, Barros-Filho B, de Oliveira M, Andrade-Neto M, de Mattos M, Mafezoli J, Pirani J (2005). ^1^H and ^13^C NMR spectra of 3,8-dimethoxyfuro[3,2-g] coumarin and maculine from *Esenbeckia grandiflora* Martius (Rutaceae). Magn Reson Chem.

[CR41] Ouete JL, Sandjo LP, Kapche DW, Liermann JC, Opatz T, Simo IK, Ngadjui BT (2013). A new flavone from the roots of *Milicia excelsa* (Moraceae). Z Naturforsch C.

[CR42] Ouete JL, Sandjo LP, Kapche DW, Yeboah SO, Mapitse R, Abegaz BM, Opatz T, Ngadjui BT (2014). Excelsoside: a new benzylic diglycoside from the leaves of *Milicia excelsa*. Z Naturforsch C.

[CR43] Rawat P, Kumar M, Sharan K, Chattopadhyay N, Maurya R (2009). Ulmosides A and B: flavonoid 6-C-glycosides from *Ulmus wallichiana*, stimulating osteoblast differentiation assessed by alkaline phosphatase. Bioorg Med Chem Lett.

[CR44] Rice LB (2006). Unmet medical needs in antibacterial therapy. Biochem Pharmacol.

[CR45] Saleem M, Nazir M, Ali MS, Hussain H, Lee YS, Riaz N, Jabbar A (2010). Antimicrobial natural products: an update on future antibiotic drug candidates. Nat Prod Rep.

[CR46] Sandjo LP, Kuete V, Tchangna RS, Efferth T, Ngadjui BT (2014). Cytotoxic benzophenanthridine and furoquinoline alkaloids from *Zanthoxylum buesgenii* (Rutaceae). Chem Cent J.

[CR47] Seukep JA, Fankam AG, Djeussi DE, Voukeng IK, Tankeo SB, Noumdem JA, Kuete AH, Kuete V (2013). Antibacterial activities of the methanol extracts of seven Cameroonian dietary plants against bacteria expressing MDR phenotypes. Springerplus.

[CR48] Shi J, Zhang X, Jiang H (2010). 2-(penta-1,3-diynyl)-5-(3,4-dihydroxybut-1-ynyl)thiophene, a novel NQO1 inducing agent from *Echinops grijsii* Hance. Molecules.

[CR49] Touani FK, Seukep AJ, Djeussi DE, Fankam AG, Noumedem JA, Kuete V (2014). Antibiotic-potentiation activities of four Cameroonian dietary plants against multidrug-resistant Gram-negative bacteria expressing efflux pumps. BMC Complement Altern Med.

[CR50] Tran QT, Mahendran KR, Hajjar E, Ceccarelli M, Davin-Regli A, Winterhalter M, Weingart H, Pages JM (2010). Implication of porins in beta-lactam resistance of *Providencia stuartii*. J Biol Chem.

[CR51] Wang D, Li F, Jiang Z (2001). Osteoblastic proliferation stimulating activity of *Psoralea corylifolia* extracts and two of its flavonoids. Planta Med.

[CR52] Wiedemann B, Lerche H, Lotter H, Neszmelyi A, Wagner H, Müller A (1999). Two novel triterpenoids from the stemwood of *Herrania cuatrecasana*. Phytochemistry.

